# Ageing leads to selective type II myofibre deterioration and denervation independent of reinnervative capacity in human skeletal muscle

**DOI:** 10.1113/EP092222

**Published:** 2024-10-28

**Authors:** Oscar Horwath, Marcus Moberg, Sebastian Edman, Andrew Philp, William Apró

**Affiliations:** ^1^ Department of Physiology, Nutrition and Biomechanics The Swedish School of Sport and Health Sciences Stockholm Sweden; ^2^ Department of Physiology and Pharmacology Karolinska Institutet Stockholm Sweden; ^3^ Department of Women's and Children's Health Karolinska Institutet Stockholm Sweden; ^4^ Centre for Healthy Ageing Centenary Institute Sydney NSW Australia; ^5^ School of Sport, Exercise and Rehabilitation Sciences University of Technology Sydney Sydney NSW Australia; ^6^ School of Sport, Exercise and Rehabilitation Sciences University of Birmingham Birmingham UK; ^7^ Department of Clinical Science, Intervention and Technology Karolinska Institutet Stockholm Sweden

**Keywords:** ageing, human skeletal muscle, NCAM, Pax7, sarcopenia

## Abstract

Age‐related loss of muscle mass and function is underpinned by changes at the myocellular level. However, our understanding of the aged muscle phenotype might be confounded by factors secondary to ageing per se, such as inactivity and adiposity. Here, using healthy, lean, recreationally active, older men, we investigated the impact of ageing on myocellular properties in skeletal muscle. Muscle biopsies were obtained from young men (22 ± 3 years, *n* = 10) and older men (69 ± 3 years, *n* = 11) matched for health status, activity level and body mass index. Immunofluorescence was used to assess myofibre composition, morphology (size and shape), capillarization, the content of satellite cells and myonuclei, the spatial relationship between satellite cells and capillaries, denervation and myofibre grouping. Compared with young muscle, aged muscle contained 53% more type I myofibres, in addition to smaller (−32%) and misshapen (3%) type II myofibres (*P *< 0.05). Aged muscle manifested fewer capillaries (−29%) and satellite cells (−38%) surrounding type II myofibres (*P *< 0.05); however, the spatial relationship between these two remained intact. The proportion of denervated myofibres was ∼2.6‐fold higher in old than young muscle (*P *< 0.05). Aged muscle had more grouped type I myofibres (∼18‐fold), primarily driven by increased size of existing groups rather than increased group frequency (*P *< 0.05). Aged muscle displayed selective deterioration of type II myofibres alongside increased denervation and myofibre grouping. These data are key to understanding the cellular basis of age‐related muscle decline and reveal a pressing need to fine‐tune strategies to preserve type II myofibres and innervation status in ageing populations.

## Abstract (highlights)

- **What is the central question of this study?**
  How does ageing impact type I and type II myofibre properties in human muscle?
- **What are the main findings and their importance?**
  Type II myofibres in healthy, recreationally active, older adults were smaller, misshapen, and had fewer satellite cells and capillaries in comparison to young adults. Older adults also manifested an increased proportion of denervated and grouped myofibres. Our data suggest that age‐related changes in skeletal muscle are highly confined to type II myofibres. These observations are key to understanding how ageing impacts skeletal muscle in the absence of inactivity, adiposity and medications.

## 1 INTRODUCTION

One of the distinct features of ageing is the progression of sarcopenia, a term describing the gradual loss of muscle mass and function that begins in the fourth decade of life (Cruz‐Jentoft et al., 2019; Janssen et al., 2000). The rate of sarcopenia progression can be accentuated by negative lifestyle and clinical factors (Lazarus et al., 2019). For instance, it is well documented that a sedentary lifestyle accelerates muscle loss and metabolic decline in ageing populations (Breen et al., 2013; Smeuninx et al., 2021) and that excess adiposity predisposes older adults to negative health and functional outcomes (Koster et al., 2011). Another factor that can complicate studies of ageing muscle is medications used by older adults, with some drugs having a beneficial impact on the regulation of muscle mass and function (e.g., ibuprofen; Trappe et al., 2011), whereas others have the opposite effect (e.g., metformin; Walton et al., 2019). Thus, to study the biological effect of ageing per se, participants across ages should be matched for health status, medications, activity levels and body composition.

At the myocellular level, several different histological features are thought to represent the ageing muscle phenotype, that is, shifts in myofibre type composition, accumulation of atrophic and angular myofibres and loss of capillarization (Andersen, 2003; Granic et al., 2023). However, many inconsistencies exist in the literature, and it is still debated whether these hallmarks of ageing are caused by ageing itself or induced secondary to an ageing lifestyle (St‐Jean‐Pelletier et al., 2017; Zampieri et al., 2015). This process is further complicated by the fact that some of these hallmarks are dependent on sex or muscle group and are apparent only at later stages of the ageing process (Aniansson et al., 1986; Karlsen et al., 2019; Roberts et al., 2018). Furthermore, muscle loss with age is not homogeneous with respect to different myofibre types, with fast myofibres being affected preferentially, whereas slow myofibres can remain unchanged until the ninth decade of life (Grosicki et al., 2022; Karlsen et al., 2019; Snijders, Nederveen, Joanisse et al., 2017). The last point highlights the importance of performing analyses separately for type I and type II myofibres to gain a better understanding of this phenotypic trait of ageing muscle.

Ageing is associated with a reduction in tissue stem cells, which in skeletal muscle are represented by the satellite cells. These cells are central for maintaining muscle homeostasis because they either fuse with existing myofibres to repair partial injuries or fuse with each other to regenerate *de novo* myofibres in the event of severe damage (Lepper et al., 2011; Murach et al., 2021). Moreover, the involvement of satellite cells in the pathophysiology of sarcopenia goes beyond declining numbers because the niche in which the satellite cells reside is also compromised with ageing (Snijders & Parise, 2017). This includes changes in the composition of the extracellular matrix, increased collagen deposition and reduced delivery of systemic factors through the microvasculature (Garg & Boppart, 2016; Nederveen et al., 2020; Snijders et al., 2014). Fortunately, the satellite cell pool retains much of its plasticity throughout life and can be preserved even in the eighth decade of life in physically active older adults (Soendenbroe et al., 2022).

A major determinant of age‐induced muscle loss is denervation, resulting from a loss of α‐motor neurons in the spinal cord and/or disruption at the muscle‐nerve site (Oda, 1984; Tomlinson & Irving, 1977). Once myofibres have lost their innervation, they start to undergo atrophy, and if this continues, they can become permanently degraded (Jones et al., 2022; Soendenbroe et al., 2021). However, during this process, the majority of myofibres are rescued by a neighbouring motor neuron in a process known as reinnervation (Jones et al., 2022; Kelly et al., 2018; Sonjak et al., 2019). Myofibre reinnervation is an important compensatory mechanism that separates well‐functioning older adults from individuals affected by sarcopenia (Piasecki et al., 2018). Our understanding of the denervation–reinnervation cycles in human muscle is, however, not yet complete, and studies addressing this using a combination of histological analyses and molecular markers remain scarce (Sonjak et al., 2019).

To date, much of our understanding of skeletal muscle across the lifespan has been generated in older adults with confounding comorbidities or in well‐functioning, highly trained healthy older adults (i.e., master athletes; McKendry et al., 2020), neither of which allow assessment of the influence of healthy ageing per se on sarcopenia. With this in mind, we used a cross‐sectional design to study a wide range of myofibre properties in two groups of healthy, lean and physically active men. Here, we focused on several different aspects of ageing muscle, such as myofibre morphology, the capillary network, satellite cell abundance and their relationship with the microvasculature, in addition to the muscle innervation profile, using a variety of histological and molecular markers. Considering the health status and activity level of the older cohort, this would allow us independently to assess the involvement of age on sarcopenia progression.

## 2 MATERIALS AND METHODS

### 2.1 Ethical approval

This study was approved by the West Midlands – Black Country Research Ethics Committee (#17/WM/0068) and the Swedish Ethical Review Authority (#2017/2107‐31/2) and conformed to the standards set by the *Declaration of Helsinki*. All participants were informed about the nature and risks associated with their participation and signed an informed consent agreement prior to their enrolment in the study. The study was registered at ClincalTrials.gov (NCT03032757).

### 2.2 Study design and participants

The original study design and participant characteristics have been described in a parallel publication (Horwath et al., 2024). Briefly, 10 healthy young (mean ± SD; 22 ± 3 years) and 11 older men (69 ± 3 years) were recruited for this study. All participants were non‐smoking and lean (body mass index < 25 kg/m^2^). Young and old participants did not differ in terms of height (177 ± 5 vs. 179 ± 5 cm), body mass (73 ± 3 vs. 73 ± 6 kg) and body mass index (23 ± 1 vs. 23 ± 1 kg/m^2^), respectively. The participants did not use medications with known effects on muscle metabolism, although some participants in the old group consumed medications against acid reflux (*n* = 1, esomeprazole), hypertension (*n* = 1, amlodipine; and *n* = 1, losartan) and benign prostate hyperplasia (*n* = 1, finasteride). All participants were considered recreationally active because they performed various forms of physical exercise two to three times per week, but they did not participate in structured resistance exercise training. Furthermore, the old men did not show any age‐related deficits with regard to their metabolic response (insulin and glucose) and anabolic response (cell signalling and rates of muscle protein synthesis) after amino acid intake and an acute bout of resistance exercise, as reported elsewhere (Horwath et al., 2024). Given that the participants had an active lifestyle and normal body weight, a normal metabolic response to feeding, and only a few incidences of minor age‐related diseases, we considered them healthy. Before the experimental day, all participants performed a standardized maximal single‐leg knee‐extensor strength test (10‐repetition maximum (10 RM)]. As expected, the young had a greater 10 RM than the old (58 ± 6 vs. 30 ± 7 kg, *P *< 0.001), respectively.

### 2.3 Muscle biopsy sampling

Muscle samples analysed in the present study were collected as described previously (Horwath et al., 2024). Samples were collected after an overnight fast after participants had refrained from strenuous physical activity for 48 h. After administrating local anaesthesia [mepivacaine, 20 mg/mL (Carbocaine; AstraZeneca AB, Sweden)], a muscle biopsy was taken from the vastus lateralis muscle using the Bergström technique with manual suction (Ekblom, 2017). Immediately upon excision, pieces appropriate for histology were blotted free of excess blood, mounted in OCT embedding medium (Tissue‐Tek OCT compound) and frozen in isopentane chilled by liquid nitrogen. Samples were stored at −80°C until cryosectioning.

### 2.4 Immunofluorescence

Muscle cross‐sections (7 µm thick) were prepared at −21°C using a cryostat (Leica CM1950). The sections were placed on microscope glass slides (VistaVision, VWR International), allowed to air dry for 1 h at room temperature, then stored at −80°C until staining. Next, six different immunofluorescence staining protocols were applied, and most of these were adopted from previous publications from our laboratory with only small adjustments (see specific references below). The primary and secondary antibodies used and their respective species, subclasses, manufacturers and concentrations are provided in Table 1.

**TABLE 1:** Primary antibodies used for immunofluorescence.

Reactivity	Primary antibody	Species	Manufacturer	Subclass	Dilution	Concentration (µg/mL)	Secondary antibody	Dilution
MyHC‐I	BA‐F8	Mouse	DSHB	IgG2b	1:50–200	51	AF GAM 488	1:200–1000
MyHC‐II	SC‐71	Mouse	DSHB	IgG1	1:200	70	AF GAM 647	1:300
Laminin	D18	Mouse	DSHB	IgG2a	1:50–100	14	AF GAM 350	1:200–500
Dystrophin	MANDYS1	Mouse	DSHB	IgG2a	1:200	36	AF GAM 488	1:300
Pax7	199010	Mouse	Abcam	IgG1	1:300	200	AF GAM 647	1:300
CD56/NCAM	347740	Mouse	BD	IgG1	1:100	50	AF GAM 647	1:300
MyHCn	NCL‐MHCn	Mouse	Novocastra	IgG1	1:100	N/A	AF GAM 647	1:300
CD31/PECAM	JC70	Mouse	SCBT	IgG1	1:400	200	AF GAM 647	1:500
CD31/PECAM	ab28364	Rabbit	Abcam	IgG	1:100	13	AF GAR 488	1:200

Abbreviations: AF, AlexaFluor; BD, Becton and Dickinson; CD, cluster of differentiation; DSHB, Developmental Studies of Hybridoma Bank; GAM, goat anti‐mouse; GAR, goat anti‐rabbit; MHCn, neonatal myosin heavy chain; NCAM, neural cell adhesion molecule; PECAM, platelet and endothelial cell adhesion molecule; SCBT, Santa Cruz BioTechnology.

For myofibre type composition, unfixed slides were blocked in 1% normal goat serum and 0.01% Triton‐X for 30 min, and incubated in a cold room (4°C) overnight (∼16 h) with a mixture of primary antibodies for myosin heavy chain (MyHC) I, MyHC II (both isoforms) and laminin (Horwath et al., 2020). For satellite cells and myonuclei, slides were fixed for 10 min in 4% paraformaldehyde (PFA) solution, blocked for 30 min, and incubated overnight with primary antibodies against MyHC I, laminin and paired box protein 7 (Pax7) (Horwath, Moberg et al., 2021; Horwath et al., 2023). For muscle capillaries, slides were fixed for 10 min in 4% PFA, blocked for 30 min (5% normal goat serum and 0.02% Triton‐X), and incubated for 2 h at room temperature with primary antibodies against MyHC I, laminin and cluster of differentiation 31 (CD31)/platelet and endothelial cell adhesion molecule (PECAM) (anti‐mouse) (Horwath et al., 2020). For co‐immunofluorescence staining of satellite cells and capillaries, slides were fixed for 10 min in 4% PFA, blocked for 30 min, and incubated overnight with a mixture of primary antibodies against MyHC I, laminin, Pax7 and CD31/PECAM (anti‐rabbit). Of note, owing to the limited number of filter cubes available on the microscope, multiple markers had to be visualized using the same fluorescence wavelength. Specifically, MyHC I, laminin and nuclei were all visualized using the 4′,6‐diamidino‐2‐phenylindole (DAPI) filter (Ex375/28, Em460/50), and the antibodies for MyHC I and laminin therefore had to be applied in high concentrations to match the fluorescence signal emitted from the DAPI‐labelled nuclei (see Figure 4c). For molecular markers of denervation [i.e., neural cell adhesion molecule (NCAM) and neonatal myosin heavy chain (MyHCn)], slides were fixed in 1% PFA for 10 min, blocked for 1 h (5% normal goat serum and 0.01% Triton‐X), and thereafter incubated overnight with primary antibodies against either MyHC I + dystrophin + NCAM or dystrophin + MyHCn. These stainings (NCAM and MyHCn) were performed with a low percentage of fixative (1% PFA) because in‐house validation revealed a similar signal in comparison to non‐fixed tissue.

Following primary antibody incubation, slides were thoroughly washed (three times, each for 5 min) in PBS, followed by 1 h incubation at room temperature with species‐ and subclass‐specific fluorescently labelled secondary antibodies (Alexa Fluor; Invitrogen, USA). This was followed by washes in PBS before the slides were mounted with a coverglass and anti‐fade fluorescence mounting media containing DAPI or not (Invitrogen, USA).

### 2.5 Microscope and image acquisition

Stained sections were captured with a widefield fluorescence microscope (CELENA S; Logos Biosystems, South Korea) using two different objectives (×4/0.13 NA and ×10/0.3 NA) and processed with the built‐in image analysis software. The fluorescence signal was recorded with a Cy5 long‐pass filter (Ex620/60, Em665lp), an enhanced yellow fluorescent protein filter (Ex500/20, Em535/30) and a DAPI filter (Ex375/28, Em460/50). Morphological analyses and subsequent quantification were performed using ImageJ (US National Institutes of Health).

### 2.6 Morphological analyses

Myofibre type composition was determined by calculating the number of myofibres of each type (i.e., type I and type II) in the whole muscle section and expressing them as a proportion of the total count, including an average of 872 ± 583 (mean ± SD) (range, 323–2867) myofibres per sample. Myofibres that stained positive for both MyHC I and II were classified as hybrids. Myofibre cross‐sectional area was determined by manually delineating 100 myofibres (50 of each type) in sections free of freezing artefacts. These were chosen by random, but myofibres close to the edge of the sections were not included for analysis. However, this analysis was not performed on MyHC coexpressing myofibres (hybrids) because these represented only a minor fraction of the total myofibre pool. The mean myofibre cross‐sectional area was obtained by averaging individual values, and the relative frequency was obtained by distributing these into size intervals of 1000 µm^2^. Myofibre area (as a percentage) was calculated as a function of myofibre type composition and area (Horwath, Envall et al., 2021). For the assessment of ‘atrophic’ myofibres in the old group, we defined atrophic myofibres as a size represented by the first percentile in the young group (Sonjak et al., 2019), which, in the present study, corresponded to ≤2339 µm^2^ (see Figure S1). Furthermore, we assessed type I and type II myofibre shape by determining the shape factor index, which serves as a marker of the shape of an object irrespective of its size (Soendenbroe et al., 2024).

The muscle microvasculature was quantified initially at the whole‐tissue level (Charifi et al., 2004). In brief, the capillary density represents the number of capillaries in a given area of muscle tissue, expressed per square millimetre. The capillary‐to‐myofibre ratio (C/F) represents the ratio between the number of capillaries and the number of myofibres. An average of 318 ± 105 (SD) myofibres and 452 ± 141 (SD) capillaries per biopsy were included for this analysis. We also assessed myofibre type‐specific capillary indices by determining capillary contacts (CC), the capillary‐to‐myofibre ratio on an individual basis (C/F_i_), the number of capillaries in relationship to myofibre area (CAFA), the capillary‐to‐myofibre perimeter exchange index (CFPE) and the number of myofibres sharing a capillary (SF) (Charifi et al., 2004; Hepple, 1997b). These analyses were performed on 50 myofibres per biopsy.

Pax7^+^/DAPI^+^ cells residing inside the laminin border were considered satellite cells and marked together with their associated myofibre type (MyHC I positive or MyHC II negative). Satellite cells were quantified in an average of 468 ± 141 (SD) myofibres per biopsy and expressed in relationship to the number of myofibres, per unit area and per nucleus. From this staining, we also determined the content of myonuclei by counting nuclei located inside the laminin border (DAPI^+^/Pax7^−^) and expressed these in relationship to the number of myofibres and the myofibre area (i.e., myonuclear domain). Myonuclei not located in the periphery of the myofibre were classified as ‘centralized nuclei’ and used as a marker of muscle regenerative history.

The spatial relationship between satellite cells and the microvasculature was assessed in a subset of muscle biopsies (owing to the limited amount of tissue) from eight young and six old participants, in accordance with procedures outlined by Nederveen et al. (2016). In brief, the distance from the satellite cell to its nearest capillary was measured by manually tracing the laminin border using the freehand tool in ImageJ. To standardize this procedure, we used the centre of the satellite cell as a starting point and measured until the border of the capillary was reached (see Figure 4c). In the event that two capillaries were located at a similar distance from the satellite cell, we measured both, and the lesser of the two was used. Care was taken not to include any longitudinally oriented myofibres, and the analysis was performed in approximately half of all the satellite cells per cross‐section.

Moreover, in a subset of muscle biopsies, the proportion of myofibres expressing NCAM (*n* = 8 young vs. 9 old) and MyHCn (*n* = 8 young vs. 7 old) was calculated from their respective stainings and expressed in relationship to the total myofibre pool. The reason for a lower number of participants included in this analysis was limited tissue availability. NCAM^+^ myofibres were identified on the basis of a strong staining throughout the cytoplasm and in the event that these myofibres were extremely small they were distinguished from potential satellite cells based on the presence of a clear dystrophin border (for an example, see Figure 5c). NCAM^+^ myofibres were also examined for the coexpression of MyHC I in order to determine their myofibre type origin and expressed as a proportion of the total NCAM^+^ myofibre pool. The analysis of NCAM and MyHCn included an average of 854 ± 578 (SD) and 844 ± 616 (SD) myofibres per biopsy, respectively.

Myofibre grouping was assessed in whole muscle cross‐sections, and the analysis was first performed in accordance with the method presented by Jennekens et al. (1971), hereafter referred to as method 1. Using this method, an ‘enclosed myofibre’ was defined as a myofibre of any type surrounded by myofibres of the same type only, and a ‘myofibre group’ was defined as a group of myofibres with at least one enclosed myofibre. Moreover, in order to strengthen our own data, we also quantified myofibre grouping in accordance with the approach described by Kelly et al. (2018), hereafter referred to as method 2. Here, two potential ‘core myofibres’ of the same type were first identified by calculating a mean number and SD of myofibres of the same type neighbouring one specific myofibre. If the number of myofibres of the same type were equal to or exceeded the previously calculated number, these were defined as real core myofibres, and all neighbouring myofibres in contact with this unit were considered grouped. This analysis contained an average of 590 ± 218 (SD) myofibres per biopsy. For each method of quantification, the proportion of grouped myofibres, the number of groups and the average group size are presented for type I and type II myofibres separately.

### 2.7 Statistical analyses

Statistical analyses were performed in GraphPad Prism (v.10.1.10 for Windows; GraphPad Software). Testing of normality was performed using the Shapiro–Wilk test and Q–Q plots, and data that did not conform to a normal distribution were either log‐transformed to meet the assumption of normality or analysed using a non‐parametric alternative (Mann–Whitney *U*‐test). Student's two‐tailed unpaired *t*‐tests were used to assess differences between young and old. Myofibre type‐specific variables were analysed using a two‐way ANOVA, with factors for age (young vs. old) and myofibre type (type I vs. type II). Significant main effects or interactions were followed up with Bonferroni's *post hoc* test. Correlational analyses were conducted using the Pearson product–moment correlation coefficient (*r*). Statistical significance was accepted at *P* ≤ 0.05. All data are presented as means ± SD or means with individual values.

## 3 RESULTS

### 3.1 Myofibre type composition and morphology

**FIGURE 1:**
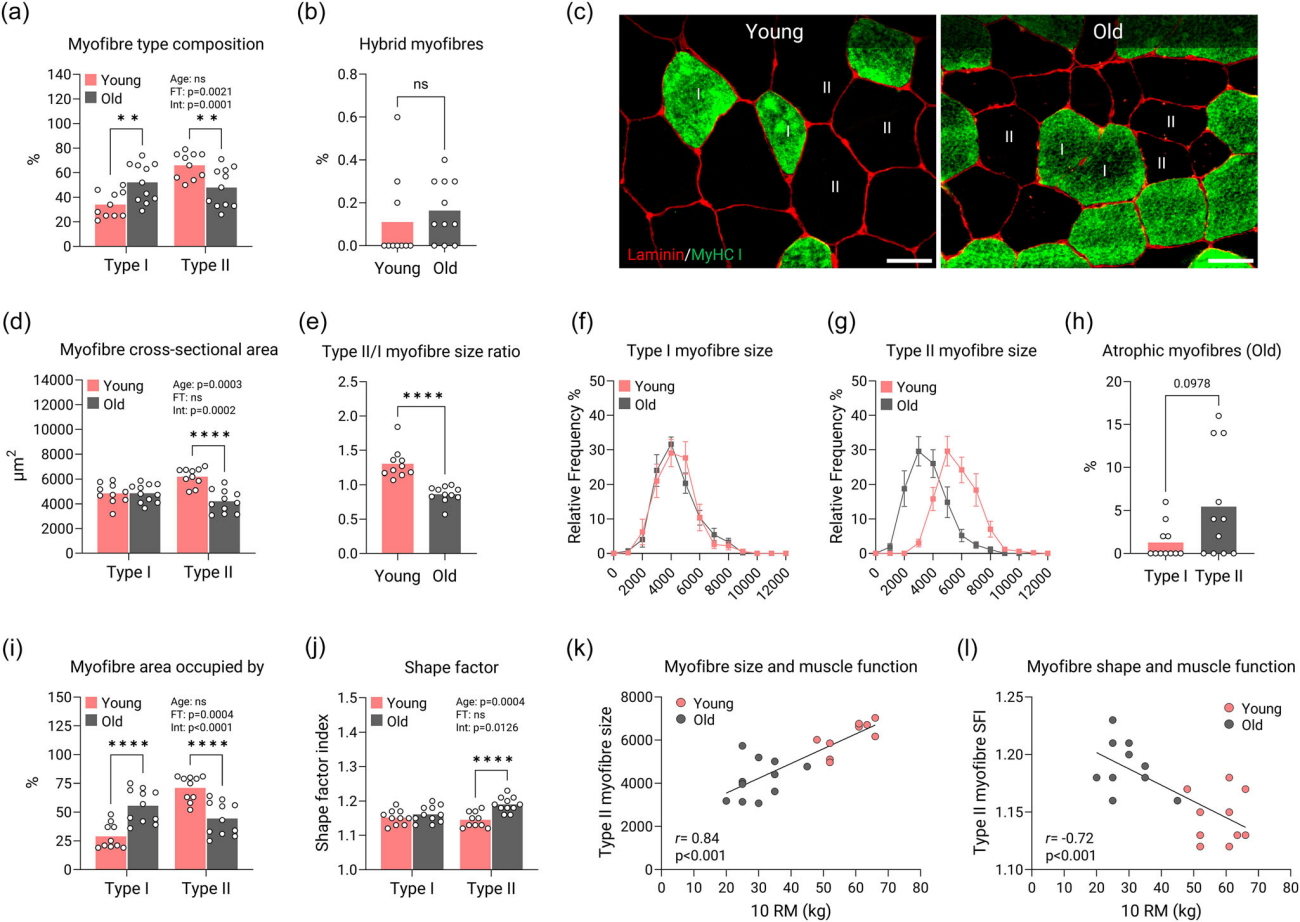
Myofibre morphology. (a) Myofibre type composition. (b) MyHC coexpressing myofibres (hybrids). (c) An example of type I (green) and type II (black) myofibre size and shape in young (left) and older adults (right). Scale bar: 50 µm. Images have been contrasted for illustrative purposes. (d) Myofibre cross‐sectional area. (e) Type II/I myofibre size ratio. (f,g) Type I myofibre size (f) and type II myofibre size (g) illustrated as frequency histograms. (h) The percentage of atrophic myofibres in the old group. (i) Area occupied by different myofibre types expressed as a percentage. (j) Shape factor index. (k, l) Correlational analyses of muscle function and type II myofibre size and shape, respectively. Data are shown as means (bars) and individual values (circles) or means ± SD. *n* = 10 young and *n* = 11 old. Some data points in (k) (*n* = 1) and (l) (*n* = 4) are masked owing to overlap. Data were analysed using a two‐way ANOVA except for (b) and (e) (Student's unpaired *t*‐tests) and (h) (Mann–Whitney *U*‐test). An effect significantly different from young is indicated by ^**^ *P *< 0.01 and ^****^ *P *< 0.0001. Abbreviations: Age, main effect of age; FT, main effect of myofibre type; Int, age × myofibre type interaction; MyHC, myosin heavy chain; ns, not significantly different; RM, repetition maximum; SFI, shape factor index.

Old muscle displayed a greater proportion of type I myofibres than young muscle (34% ± 11% in young vs. 52% ± 16% in old; *P* = 0.0001; Figure 1a). This was probably not related to ongoing myofibre transitions because the two groups displayed a similar proportion of hybrid myofibres (<0.5% for both groups; Figure 1b). Type I myofibre size was similar between groups (young, 4846 ± 817 µm^2^; old, 4861 ± 744 µm^2^), whereas type II myofibre size was ∼32% lower in old muscle (young, 6197 ± 713 µm^2^; old, 4201 ± 906 µm^2^; *P* = 0.0001; Figure 1c,d). As a result, the type II‐to‐type I myofibre size ratio was ∼30% lower in the old group (*P *< 0.0001; Figure 1e). Old muscle also displayed a clear leftward shift in the frequency histogram specifically for type II myofibres (Figure 1f,g). This coincided with the observation that myofibre size variation was greater in old compared with young muscle specifically in type II myofibres (coefficient of variation, 23.8% ± 3.8% and 17.8% ± 4.6%, respectively; *P* = 0.0053; Table S1). The proportion of atrophic type II myofibres tended to be higher than that of type I myofibres in the old group (5.5% ± 6.3% and 1.3% ± 2.1%, respectively; *P* = 0.0978; Figure 1h). Collectively, aged muscle was occupied to a greater extent by type I myofibre area in comparison to young muscle (29% ± 11% in young vs. 55% ± 14% in old; *P* = 0.0001; Figure 1i). Myofibre shapes in young and old muscles were similar for type I myofibres, whereas the old muscles had ∼3% higher shape factor index than young muscles in their type II myofibres (*P *< 0.0001; Figure 1j). Correlational analyses revealed that type II myofibre size was positively correlated with 10 RM load (*r* = 0.84, *P *< 0.001; Figure 1k) and that type II myofibre shape was negatively correlated with 10 RM load (*r* = 0,72, *P *< 0.001; Figure 1l). However, no significant correlations were found for type I myofibres (Figure S2a,b).

### 3.2 Muscle capillarization

**FIGURE 2:**
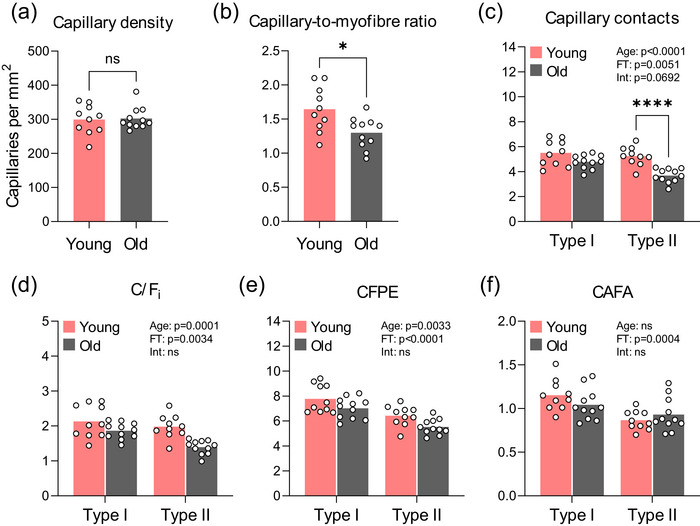
Microvascular indices: (a) capillary density; (b) capillary‐to‐myofibre ratio; (c) capillary contacts; (d) C/F_i_; (e) CFPE; and (f) CAFA. Data are shown as means (bars) and individual values (circles). *n* = 10 young and *n* = 11 old. All data were analysed using a two‐way ANOVA except for (a) and (b), which were compared using Student's unpaired *t*‐tests. An effect significantly different from young is indicated by ^*^ *P *< 0.05 and ^****^ *P *< 0.0001. Abbreviations: Age, main effect of age; CAFA, the number of capillaries in relationship to myofibre area; C/F_i_, capillary to myofibre ratio on an individual basis; CFPE, capillary‐to‐myofibre perimeter exchange index; FT, main effect of myofibre type; Int, age × myofibre type interaction; ns, not significantly different.

Whole‐muscle capillary density was similar in old and young muscles (Figure 2a), whereas older adults showed ∼21% lower capillary‐to‐myofibre ratio compared with young adults (*P* = 0.0113; Figure 2b). In contrast, CC tended (interaction effect *P* = 0.0692) to be lower by ∼29% in old compared with young muscle, specifically in type II myofibres (*post hoc* test *P *< 0.0001; Figure 2c). A similar pattern was observed for the individual capillary‐to‐myofibre ratio (C/F_i_) and the capillary‐to‐myofibre perimeter exchange index (CFPE), revealing main effects for age and myofibre type, but none of these displayed a significant interaction effect (Figure 2d,e). The number of capillaries in relationship to myofibre area (CAFA) revealed a main effect of myofibre type, whereby type II myofibres had ∼18% lower values compared with type I myofibres, irrespective of age (Figure 2f). No main effects of either age or myofibre type was observed for the number of myofibres sharing a capillary (data not illustrated).

### 3.3 Satellite cells, myonuclei and myonuclear domains

**FIGURE 3:**
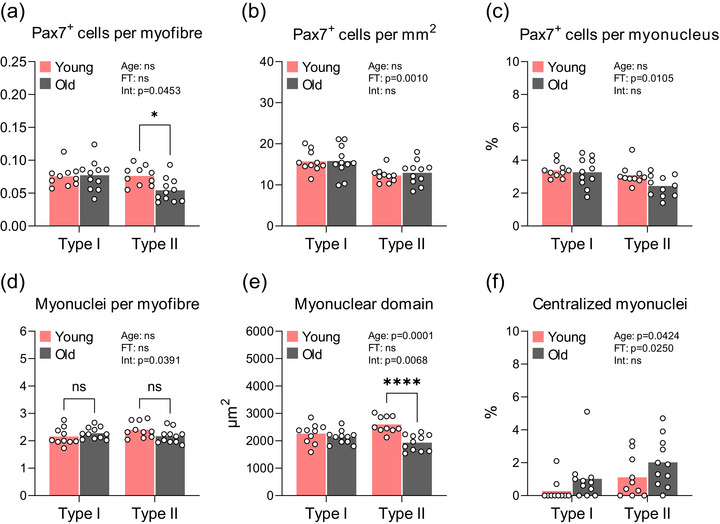
Satellite cell and myonuclei content: (a) Pax7^+^ cells per myofibre; (b) Pax7^+^ cells per square millimetre; (c) Pax7^+^ cells per myonucleus; (d) myonuclei per myofibre; (e) myonuclear domain; and (f) proportion of centralized myonuclei. Data are shown as means (bars) and individual values (circles). *n* = 10 young and *n* = 11 old. All data were analysed using a two‐way ANOVA. An effect significantly different from young is indicated by ^*^ *P *< 0.05 and ^****^ *P *< 0.0001. Abbreviations: Age, main effect of age; FT, main effect of myofibre type; Int, age × myofibre type interaction; ns, not significantly different; Pax7, paired box protein 7.

Old muscle had fewer satellite cells associated with type II myofibres than young muscle (0.05 and 0.08 satellite cells per myofibre, respectively; *P* = 0.0186; Figure 3a), whereas the number of satellite cells associated with type I myofibres was similar between groups. However, when satellite cells were expressed in relationship to tissue area or per myonucleus, these differences were no longer apparent and only a main effect of myofibre type was observed (Figure 3b,c). Old muscle did not show alterations in the content of myonuclei in either of the myofibre types (Figure 3d) but displayed a 26% smaller myonuclear domain specifically in type II myofibres as a result of declining myofibre size (*P *< 0.0001; Figure 3e). In general, the proportion of centralized myonuclei was higher in old compared with young muscle (*P* = 0.0424) and higher in type II compared with type I myofibres (*P* = 0.0250), but no interaction effect was observed (Figure 3f).

### 3.4 Relationship between satellite cells and capillaries

**FIGURE 4:**
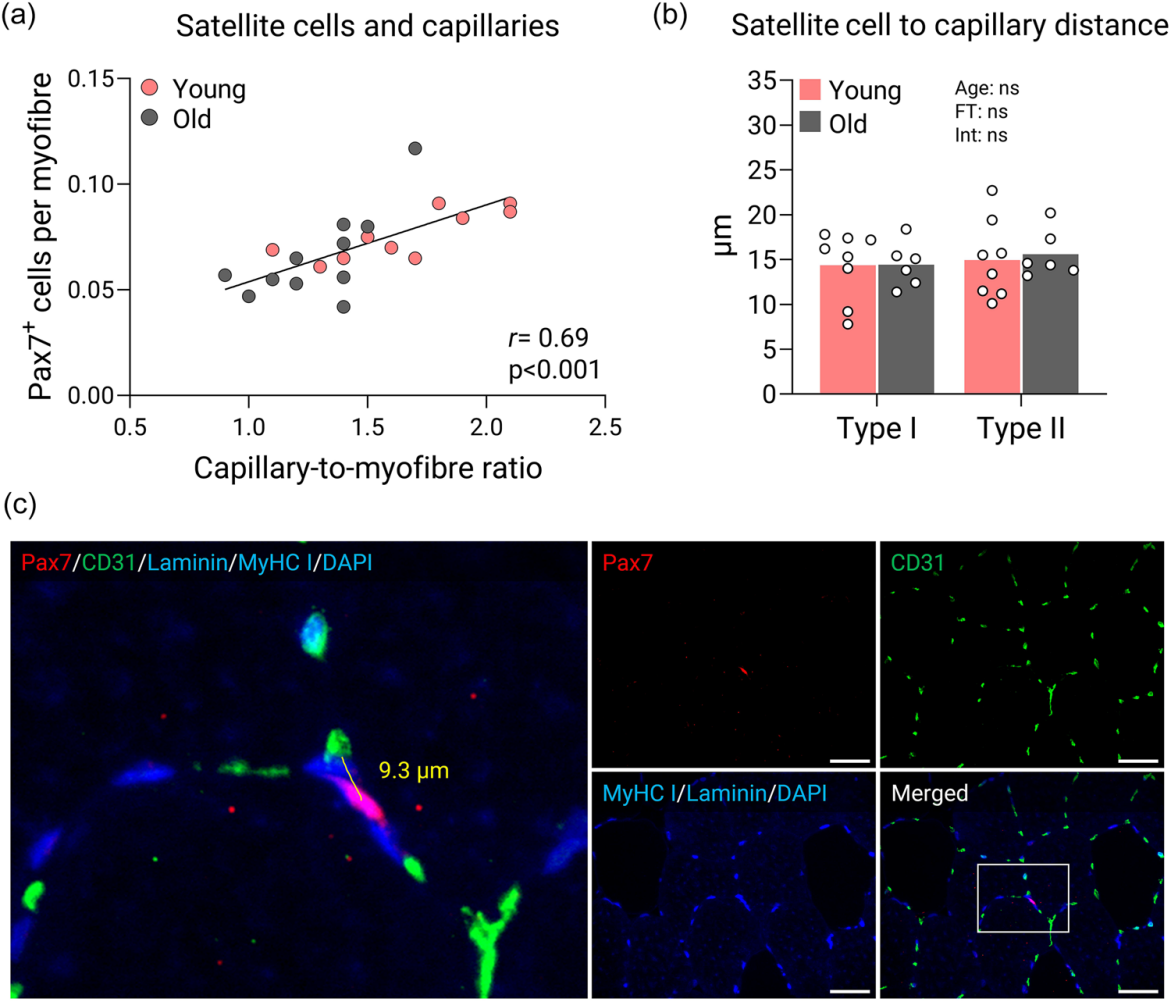
Relationships between satellite cells and capillaries: (a) correlational analyses of satellite cell and capillary content; (b) satellite cells and the distance to nearest capillary analysed using a two‐way ANOVA; and (c) example of co‐immunofluorescence staining of satellite cells, capillaries, laminin, MyHC I and myonuclei (left panel). The distance between a single satellite cell and its nearest capillary is highlighted in yellow. Single filter images are shown of satellite cells (top middle panel), capillaries (top right panel), type I myofibres, laminin and myonuclei (bottom middle panel) and a merged image (bottom right panel). Scale bars: 50 µm. Images have been contrasted for illustrative purposes. Data are shown as means (bars) and individual values (circles). *n* = 10 young and *n* = 11 old for correlational analyses; and *n* = 8 young and *n* = 6 old for satellite cell‐to‐capillary distance. Abbreviations: CD31, cluster of differentiation 31; MyHC, myosin heavy chain; Pax7, paired box protein 7.

The number of satellite cells in a mixed myofibre population was positively correlated with capillary‐to‐myofibre ratio (*r* = 0.69, *P *< 0.001; Figure 4a). This led us to investigate this relationship further by quantifying the spatial distance between satellite cells and their nearest capillary. However, this analysis revealed that satellite cells were located at a similar distance from their nearest capillary irrespective of age and myofibre type (Figure 4b).

### 3.5 Muscle innervation profile

**FIGURE 5:**
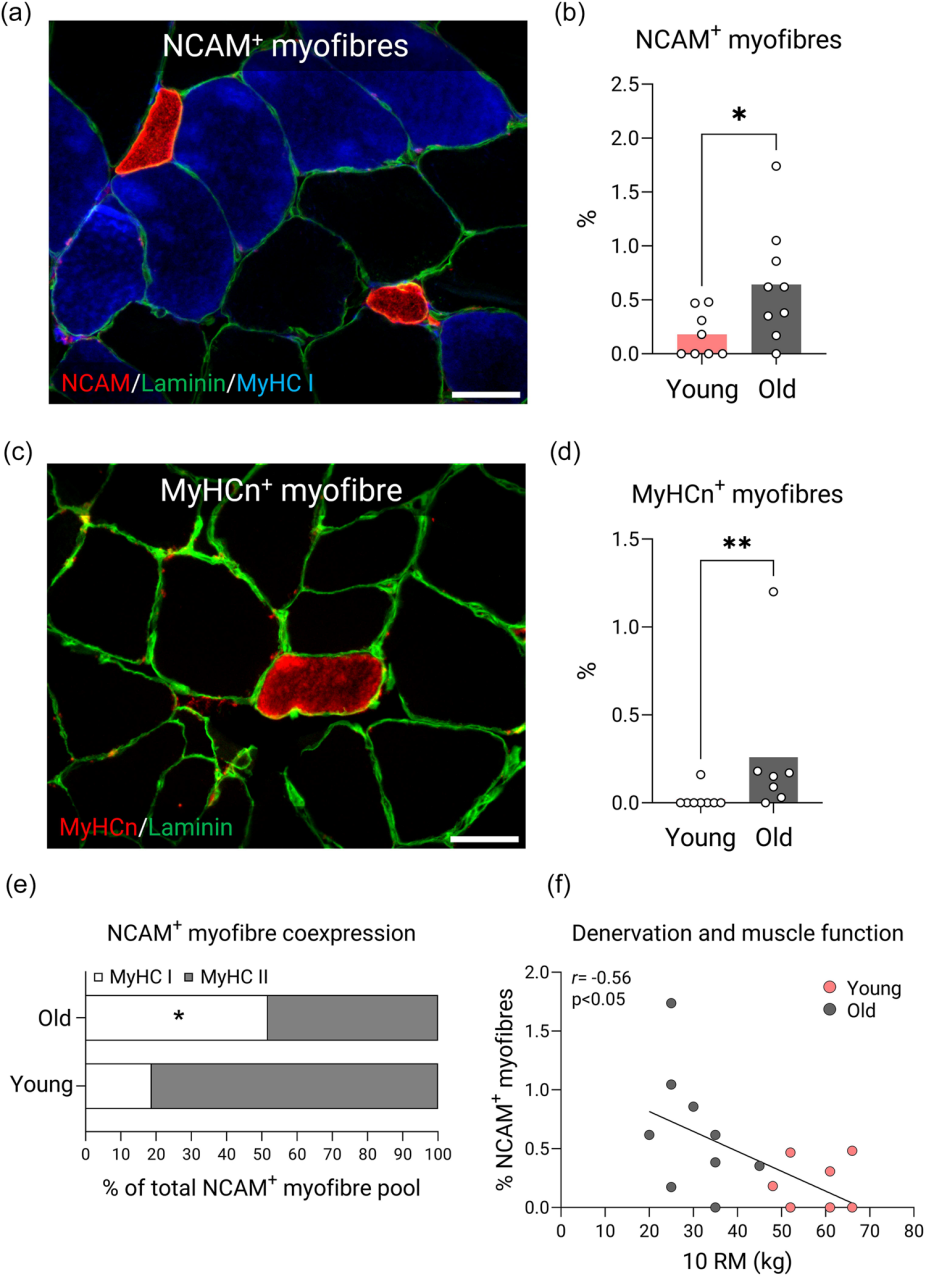
Molecular markers of denervation. (a) Example of a NCAM^+^ myofibres (red) along with the basal lamina (green) and type I myofibres (blue) in an older adult aged 72 years. (b) Proportion of NCAM^+^ myofibres in young and older adults compared using Student's unpaired *t*‐test (*n* = 8 young and *n* = 9 old). (c) Example of an MyHCn^+^ myofibre (red) along with the basal lamina (green) in an older adult aged 72 years. (d) Proportion of MyHCn^+^ myofibres in young and older adults compared using a Mann–Whitney *U*‐test (*n* = 8 young and *n* = 7 old). (e) Coexpression of NCAM^+^ myofibres, revealing the proportion of NCAM^+^ myofibres that express either MyHC I or II in old (upper bar) and young (lower bar) adults. (f) Correlational analyses of NCAM^+^ myofibres and muscle function. Scale bars: 50 µm. Images have been contrasted for illustrative purposes. Data are shown as means (bars) and individual values (circles) for (b) and (d), and as means only for (e) (parts of whole). Some data points in (f) (*n* = 1) are masked owing to overlap. An effect significantly different from young is indicated by ^*^ *P *< 0.05 and ^**^ *P *< 0.01. Abbreviations: MyHC, myosin heavy chain; MyHCn, neonatal myosin heavy chain; NCAM, neural cell adhesion molecule; RM, repetition maximum.

NCAM^+^ myofibres were observed in both groups but were more abundantly expressed (2.6‐fold) in old compared with young muscles (*P* = 0.0347; Figure 5b). Old muscle also had a greater proportion of NCAM^+^ myofibres that coexpressed MyHC I compared with young muscle (51% ± 34% in old vs. 19% ± 21% in young; *P* = 0.0428; Figure 5e). Correlational analyses revealed that the proportion of NCAM^+^ myofibres was negatively correlated with 10 RM load (*r* = −0.56, *P *< 0.05; Figure 5f). MyHCn^+^ myofibres were extremely rare in young muscle samples (present only in one subject), whereas all but one subject manifested positive MyHCn staining in the old group. The proportion of MyHCn^+^ myofibres was higher in old compared with young muscles (0.26% ± 0.42% in old vs. 0.02% ± 0.06% in young; *P* = 0.0064; Figure 5f). Representative images of myofibres expressing NCAM and MyHCn are presented in Figure 5a,c, respectively.

### 3.6 Myofibre type grouping

**FIGURE 6:**
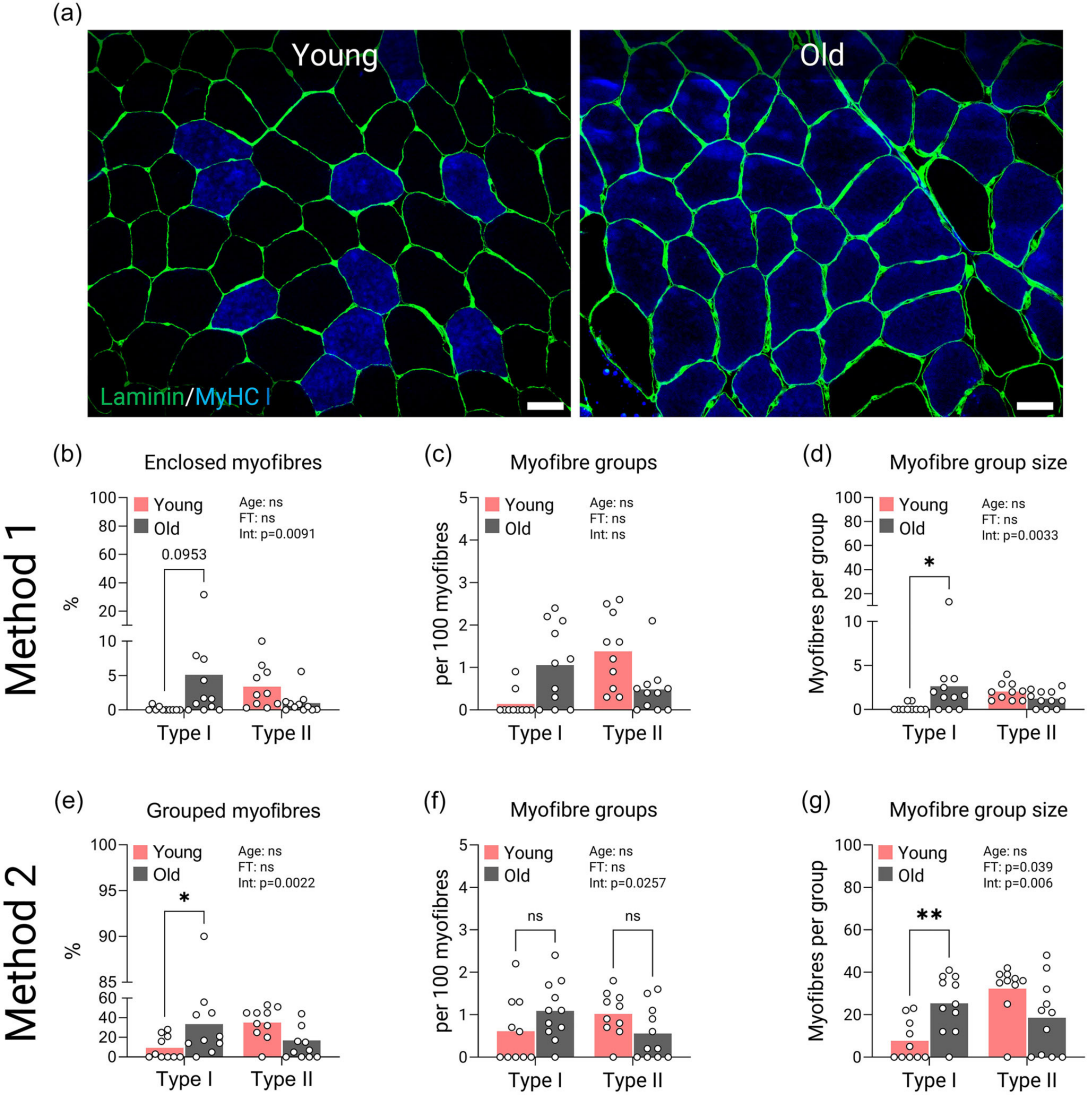
Myofibre type grouping. (a) Example of myofibre type grouping in type I myofibres (blue) and type II myofibres (black) in young (left) and older adults (right). Scale bar: 50 µm. Images have been contrasted for illustrative purposes. (b) Enclosed myofibres. (c) Number of myofibre groups. (d) Myofibre group size. (e) Grouped myofibres. (f) Number of myofibre groups. (g) Myofibre group size. Method 1 and 2 refers to the two different methods used to determine myofibre type grouping, as described in the Materials and methods section. Data are shown as means (bars) and individual values (circles). *n* = 10 young and *n* = 11 old. All data were analysed using a two‐way ANOVA. An effect significantly different from young is indicated by ^*^ *P *< 0.05 and ^**^ *P *< 0.01. Abbreviations: Age, main effect of age; FT, main effect of myofibre type; Int, age × myofibre type interaction; ns, not significantly different.

On the basis of method 1, the proportion of grouped type I myofibres tended to be higher for old compared with young muscles (5.1% ± 9.1% vs. 0.1% ± 0.3%; *post hoc* test *P* = 0.0953; Figure 6b). However, this was not driven by an increased number of myofibre groups (Figure 6c), but by an increased myofibre group size (*P* = 0.0033; Figure 6d). A similar pattern was observed when applying method 2, showing that the proportion of grouped type I myofibres was higher in old than in young muscles (33% ± 28% vs. 9% ± 12%; Figure 6e), with no differences seen in the number of myofibre groups (Figure 6f), but myofibre group size was higher in old than in young muscles (*P* = 0.006; Figure 6g). Representative images of myofibre type grouping in young and old muscle cross‐sections are presented in Figure 6a.

## 4 DISCUSSION

Our understanding of the biological effect of ageing is often limited by confounding factors, such as age‐related changes in physical activity, body composition and use of various medications. In an attempt to overcome this issue, we performed a comprehensive immunohistological assessment of skeletal muscle obtained from healthy, lean and recreationally active young and older adults. By comparing these cohorts, we report that muscle from healthy older adults demonstrates a greater proportion of type I myofibres, marked deficits in type II myofibre morphology, and a selective loss of capillaries and stem cells associated with type II myofibres. Aged muscle also displays indications of myofibre denervation alongside type I myofibre grouping, which together suggests ongoing cycles of denervation–reinnervation.

### 4.1 Myofibre type composition and hybrid myofibres

Despite decades of research, it is still debated whether myofibre type composition in human muscle changes with age (Andersen, 2003). This discussion is complicated by variations in study population and design, sampling site and methodology (Larsson et al., 1978; Lexell et al., 1988; Frontera et al., 2000; St‐Jean‐Pelletier et al., 2017). Although we cannot rule out the possibility of a genetic component, our results indicate that ageing shifts muscle towards a type I myofibre‐dominant phenotype, as evident by a greater proportion of slow myofibres in old compared with young muscle (52% vs. 34%). Intriguingly, the type I myofibre‐dominant phenotype in the old cohort was not associated with an increased proportion of myofibres coexpressing MyHC I and II, with the overall contribution of hybrids to the total myofibre pool being extremely low (<0.5%). Our data therefore suggest that ongoing transitions from fast to slow myofibres play a minor role in maintaining the present phenotype, yet we cannot rule out that a transitory phase has already been completed during earlier years. Alternatively, the slow phenotype seen here might stem from a preferential loss of large motor units, ultimately leading to a reduction of the type II myofibre pool. This, however, remains uncertain, because direct counting of the total myofibre pool using autopsy material has proposed that type I and type II myofibres are lost to the same extent in human muscle (Lexell et al., 1988).

### 4.2 Myofibre size and shape

In the present study, we observed marked type II myofibre atrophy with ageing (−32%), whereas type I myofibre size was highly preserved, thereby adding to the growing body of literature implying that fast myofibres are affected disproportionately during the ageing process (Larsson et al., 1978; Verdijk et al., 2007; Karlsen et al., 2019; Grosicki et al., 2022). This effect was further underlined as type II myofibres displayed a clear leftward shift in the frequency histogram, and more were classified as atrophic (i.e., <2339 µm^2^). From a functional perspective, a change of this magnitude is expected to have dire consequences for overall muscle function given that explosive contractions rely on a functioning pool of fast myofibres (Aagaard & Andersen, 1998). This might, for example, negatively impact the ability to perform activities of daily living, such as walking upstairs or counterbalancing falls. This relationship was reinforced by our analyses, in which type II myofibre size was correlated well with 10 RM muscle strength, whereas a similar relationship did not exist for type I myofibres. This finding aligns well with the notion that type II myofibre atrophy is a major determinant of muscle weakness in older adults (Nilwik et al., 2013).

In the present study, all subjects were considered recreationally active, meaning that they engaged in different forms of physical activity two to three times per week, although they did not perform any structured resistance exercise training. Despite this, we observed clear signs of type II myofibre atrophy in the older cohort, which indicates that a generally active lifestyle is not sufficient for preserving fast myofibre size in older adults. This is supported by recent data showing that lifelong exercising older adults, recruited specifically on the basis of performing sports that would ensure recruitment of fast myofibres, still demonstrated type II myofibre atrophy in comparison to a young control group (Soendenbroe et al., 2022). In fact, the type II myofibre atrophy experienced by these active individuals was comparable to that of age‐matched inactive controls (Soendenbroe et al., 2022). Moreover, selective type II myofibre atrophy has been documented in endurance‐ and sprint‐trained master athletes (Klitgaard et al., 1990; Korhonen et al., 2006; McKendry et al., 2020). This indicates that fast myofibre atrophy is an age‐related phenomenon that is independent of activity level. It is also striking that some exercise modalities (e.g., running and cycling) seem to have little impact on attenuating type II myofibre atrophy, even when practised at a relatively high frequency by those with life‐long experience. In contrast, performing systematic resistance exercise training is efficient for preserving fast myofibre size, because strength‐trained master athletes demonstrated similar or even superior type II myofibre areas compared with young individuals (Klitgaard et al., 1990; Toien et al., 2023).

In the present study, we also assessed myofibre shape, because this was recently proposed as an independent predictor of muscle mass in ageing populations (Soendenbroe et al., 2024). In line with previous work, we found that type II myofibres in aged muscle were misshapen compared with young muscle, whereas a similar pattern was not observed for the type I myofibres. This was found in parallel with an inverse relationship between type II myofibre shape and muscle strength, whereas again, no such relationship was seen for slow myofibres. These data further support the notion that muscle weakness in ageing populations is dependent on type II myofibre‐specific indices, with a misshapen morphology being another important factor to consider (Barnouin et al., 2017; Soendenbroe et al., 2024).

### 4.3 Muscle capillarization

The capillary network is crucial for delivering oxygen and nutrients to skeletal muscle and plays a major role for preserving glucose tolerance and in the adaptation to exercise (Snijders, Nederveen, Joanisse et al., 2017; Snijders, Nederveen, Verdijk et al., 2017). In the present study, we quantified muscle capillarization initially in a mixed myofibre population. Here, we found that although the capillary density was well maintained, aged muscle had ∼21% lower capillary‐to‐fibre ratio than young muscle. At the myofibre level, we found a selective decline in the number of capillaries surrounding type II myofibres, whereas type I myofibres were unaffected. Taken together, our results show that although some degree of microvascular alterations occur with age, the overall diffusion capacity is not reduced in muscle of healthy older adults, as indicated by the unchanged capillary density. This conclusion is supported by the fact that these older adults showed similar or even higher increase in amino acid uptake in skeletal muscle following ingestion of an amino acid‐rich drink, in comparison to young adults (Horwath *et al.*, 2024). This suggests that the capacity to accommodate blood flow through the microvasculature and thereby deliver oxygen and nutrients to skeletal muscle is preserved. It is possible that a shift towards more type I myofibres with ageing is beneficial in this regard. Furthermore, our data also show that ageing is associated with capillary rarefaction that occurs specifically in type II myofibres. Interestingly, given that the capillary rarefaction occurred in parallel with a loss of type II myofibre area, the coupling between capillary supply and myofibre area was maintained. Other studies have also shown that capillarization is positively associated with myofibre size during age‐induced atrophy (Barnouin et al., 2017), initial phases of disuse atrophy (Hendrickse et al., 2022) and hypertrophy (Holloway et al., 2018). It thus appears that the loss of capillaries is proportional to the reduction in myofibre size in ageing muscle.

### 4.4 Satellite cells and their spatial relationship with the microvasculature

Since the original discovery in rodent muscle nearly 50 years ago (Snow, 1977), several studies in humans have shown that the number of satellite cells and their function diminish with increasing age (Kadi et al., 2004; Petrella et al., 2006; Snijders et al., 2014). In the present study, we report that older adults have ∼38% fewer satellite cells associated with their type II myofibres compared with young adults, whereas no differences were seen for type I myofibres. Given the exercising habits of our older cohort, we did not expect to see such a distinct difference. It might be that retention of the satellite cell pool is highly load dependent and therefore not replenished unless frequently stimulated by high‐intensity contractions, as shown by Soendenbroe et al. (2022).

Emerging evidence suggests that muscle capillaries (i.e., endothelial cells) are crucial for maintaining adequate satellite cell function (Christov et al., 2007; Snijders, Nederveen, Joanisse et al., 2017; Nederveen et al., 2018). Anatomically, these two cell types are typically located within a close distance from each other in young muscle, whereas a larger distance has been observed in aged muscle, thereby serving as a possible mechanism for impaired satellite cell activity with increasing age (Nederveen et al., 2016). In the present study, we found that the distance between satellite cells and their nearest capillary was unaltered in aged muscle, and this was seen despite parallel declines in both variables (type II myofibres). The fact that this anatomical relationship was intact could indicate that satellite cells lost before this stage were the ones located furthest away from capillaries and/or that the satellite cells had migrated closer to existing capillaries to compensate for lesser delivery of systemic factors.

### 4.5 Myonuclei and myonuclear domains

In the present study, we did not observe any age‐related changes in the number of myonuclei. However, we observed reductions in type II myofibre size with ageing (−32%), which was not accompanied by a proportional loss of myonuclei, thus rendering a reduced myonuclear domain specifically in type II myofibres (−26%). Although these data go against the original myonuclear domain hypothesis that posited a relatively fixed relationship between the content of myonuclei and cell volume (Allen et al., 1999), this theory has been refined over the years, and it is now accepted that the myonuclear domain is less rigid than initially believed (Murach et al., 2018). Regardless, this raises the question of whether myonuclei are lost during atrophy, which has been heavily debated in recent years. Our data suggest that in these specific conditions (i.e., age‐related atrophy), there are no myonuclear losses. These findings are supported by other studies using either cross‐sections or single fibres from adults of similar ages (Verdijk et al., 2007; Battey et al., 2024). In contrast, a tendency towards myonuclear losses has been reported in older men aged 83–94 years, and given that the authors reported type II myofibre atrophy of similar magnitude to that seen here (−36%), this might suggest that ageing itself is a key driver of myonuclear losses (Karlsen et al., 2019). However, owing to the cross‐sectional nature of our study, these data should be interpreted with caution, particularly with regard to myonuclei, which might be impacted by training performed much earlier in life (Bruusgaard et al., 2010; Eftestol et al., 2022). In addition, the present study focused on the number of myonuclei, whereas emerging evidence suggests that ageing also impacts the spatial arrangement and myonuclear morphology, which might have important implications for the regulation of gene expression and protein synthesis (Cristea et al., 2010).

### 4.6 Muscle innervation profile

To investigate the impact of ageing on muscle innervation status, we applied molecular markers for denervation and used myofibre type grouping as a proxy for muscle reinnervation (Sonjak et al., 2019). Here, we showed that aged muscle had more NCAM‐ and MyHCn‐expressing myofibres than young muscle, indicating the presence of denervation. However, given that both markers represented <1% of the total pool, the relative burden of denervation appear relatively low in our ageing cohort. These data are in accordance with healthy, physically active older adults (Mosole et al., 2014; Soendenbroe et al., 2022), whereas ageing populations living a more sedentary lifestyle typically manifest higher numbers (>5%) (Sonjak et al., 2019). Collectively, this points towards a protective effect of physical activity on muscle innervation status in ageing individuals.

Furthermore, owing to the disproportionate impact on type II myofibres seen in our ageing cohort (i.e., marked myofibre atrophy and irregular shape), we sought to investigate whether this could be explained by a larger burden of denervation in this fibre type. However, given that we found a similar number of NCAM^+^ myofibres being co‐stained as type I and type II in the older group (Figure 5e), the myofibre types seem to be affected equally by denervation with ageing. In contrast, if the two myofibre types are affected to a similar extent by denervation events, as shown by the co‐staining experiment, but morphological changes occur only in one of them, this might suggest that type II myofibres have a reduced capacity to compensate for denervation events, whereas the type I myofibres are inherently more resilient to the same stimuli. Understanding the intrinsic mechanisms whereby certain myofibre types are protected against denervation would have important implications for preserving muscle mass in ageing populations. Future studies are thus warranted to address this at the single‐myofibre level.

Reinnervation is triggered as a rescuing mechanism to mitigate irreversible degradation of whole motor units following denervation (Soendenbroe et al., 2021; Jones et al., 2022). If successful, this leads to an increased pool of myofibres belonging to one specific motor unit (i.e., myofibre grouping), which is thought to be a determining factor for preventing the onset of sarcopenia (Piasecki et al., 2018). The literature on myofibre grouping is, however, not clear, because some work has shown apparent myofibre grouping in older adults (Kelly et al., 2018; Sonjak et al., 2019; Toien et al., 2023), whereas other work has not (Messa et al., 2020; Krakova et al., 2023). Much of the discrepant findings could be attributed to different ways of histological quantification of myofibre grouping. In the present study, a greater proportion of grouped type I myofibres was detected in aged compared with young muscle. This effect was driven primarily by an increased size of existing groups (i.e., more myofibres per individual group, rather than an increased number of groups per se). However, based on our data we cannot completely rule out the possibility that the number of myofibre groups also increases with ageing, because we also noted a numerical increase for this variable, particularly when applying the first method. Furthermore, the application of two separate methods of quantification, which showed a highly similar pattern, provides additional validation of our data. This is particularly important because method 2 is more conservative and accounts, at least to some degree, for the overall myofibre type composition, which represents a highly influential factor in the assessment of myofibre grouping (Kelly et al., 2018). Despite this, it is difficult to separate the impact of an altered myofibre type composition on myofibre type grouping completely, particularly given that these two processes seem to occur in parallel and are most likely to be a consequence of the same physiological event (i.e., denervation) (Jones et al., 2022). However, the fact that aged muscle displayed a clear deviation from the normal ‘checkerboard’ pattern whilst being composed of ∼50% type I myofibres provides further support to our conclusions. Therefore, it might be that more and larger groups of type I myofibres in aged muscle are a consequence of successful motor unit remodelling and thereby indicative of a preserved capacity for reinnervation in this cohort.

The present study also has limitations that warrant some consideration. First, the cross‐sectional design limits the ability to make causal inferences, and although we made an effort to recruit individuals with similar characteristics, between‐group differences could be accounted for by factors other than ageing per se (e.g., genetics). Longitudinal studies are therefore necessary to confirm these findings. Second, to gain a better understanding of the impact of living an active lifestyle at an advanced age, this study would have benefitted from inclusion of an age‐matched inactive control group. Likewise, although we carried out a comprehensive analysis of healthy aged muscle, there were only men included in the present study, and the findings may therefore not apply to women and those with advanced stages of sarcopenia. The sample size might also have limited our ability to detect smaller yet meaningful differences between the groups. Lastly, on a technical note, future studies with similar objectives should consider using a microscope with four to six different filter cubes to allow for a wider variety of fluorophore combinations.

## 5 CONCLUSION

In conclusion, based on a comprehensive histological analysis of aged muscle, our results show that ageing is associated with a type I myofibre‐dominant muscle, marked deficits in type II myofibre size and shape, a lesser abundance of stem cells and capillaries specifically surrounding type II myofibres. We also found that aged muscle had an increased proportion of denervated and grouped myofibres, which could indicate ongoing cycles of denervation–reinnervation. Our data also support the notion that type II myofibre size and shape and muscle innervation status are key determinants of muscle decline in a cohort of healthy, recreationally active, older adults. However, future studies are warranted to elucidate the molecular mechanisms behind these events in human skeletal muscle. These data have large translational value to better shape strategies to delay or reverse age‐induced muscle deterioration in ageing populations.

## AUTHOR CONTRIBUTIONS

William Apró and Andrew Philp conceived the research. Oscar Horwath, Andrew Philp and William Apró designed the research. Oscar Horwath, Marcus Moberg, Sebastian Edman and William Apró conducted the human trial. Oscar Horwath performed the immunohistochemical analyses and interpreted the data, assisted by Sebastian Edman, Marcus Moberg and William Apró. Oscar Horwath prepared the figures and drafted the manuscript. All authors read, contributed intellectually, edited, and approved the final version of the manuscript. All authors agreed to be accountable for all aspects of the work in ensuring that questions related to the accuracy or integrity of any part of the work are appropriately investigated and resolved. All persons designated as authors qualify for authorship, and all those who qualify for authorship are listed.

## CONFLICT OF INTEREST

The authors have no conflict of interest to declare.

## Supporting information




**Figure S1**. Myofibre size in young adults. (a) Type I myofibre size. (b) Type II myofibre size. (c) Mixed myofibre size. Data are illustrated as means (red line) with individual values (circles). The mixed myofibre size at the first percentile represents the cut‐off value for detecting atrophic myofibres in the group of older adults.

**Figure S2**. Correlations. Correlational analyses between muscle function and type I myofibre size (a) and shape (b). Young, *n* = 10; and old, *n* = 11. Some data points (*n* = 4) in (b) are masked owing to overlap. Abbreviations: RM, repetition maximum; SFI, shape factor index.

**Table S1**. Fibre size variation in muscle cross‐sections from young and older adults.

## Data Availability

Data are available upon reasonable request to the corresponding author.
