# Supplementary figures and images for: Ageing leads to selective type II myofibre deterioration and denervation independent of reinnervative capacity in human skeletal muscle
Source: Exp Physiol. 2024 Oct 28;110(2):277–92. doi: 10.1113/EP092222 (PMC11782179; doi:10.1113/EP092222)

# Supplementary Figure 1

**A**  
Type I myofiber size

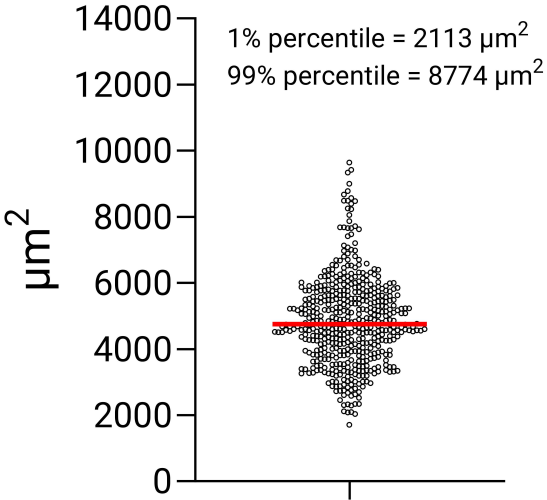

**B**  
Type II myofiber size

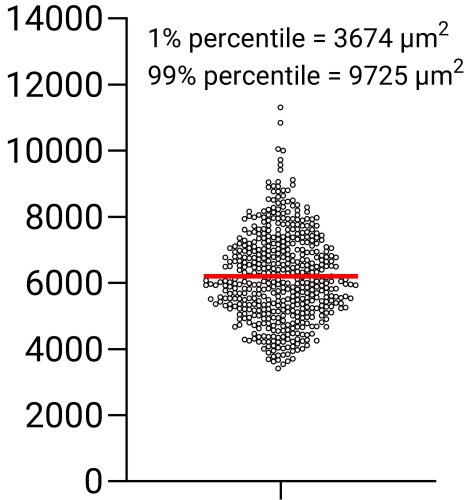

**C**  
Mixed myofiber size

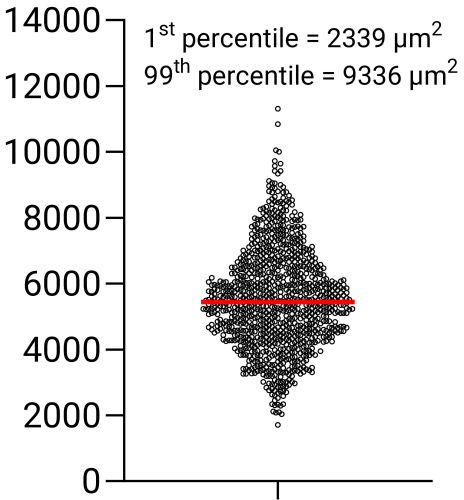

Supplement: Supplementary file 1 — Figure S1. Myofibre size in young adults. (a) Type I myofibre size. (b) Type II myofibre size. (c) Mixed myofibre size. Data are illustrated as means (red line) with individual values (circles). The mixed myofibre size at the first percentile represents the cut‐off value for detecting atrophic myofibres in the group of older adults. [file EPH-110-277-s001.pdf]

# Supplementary Figure 2

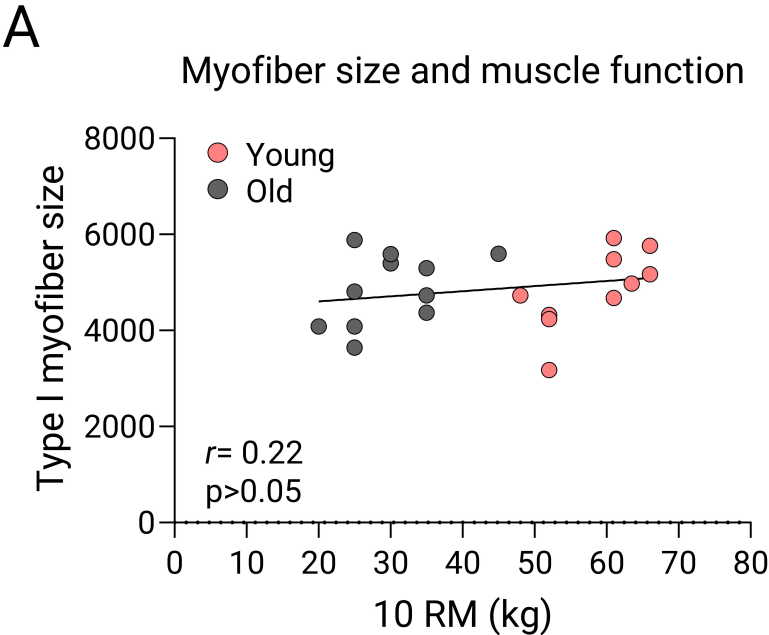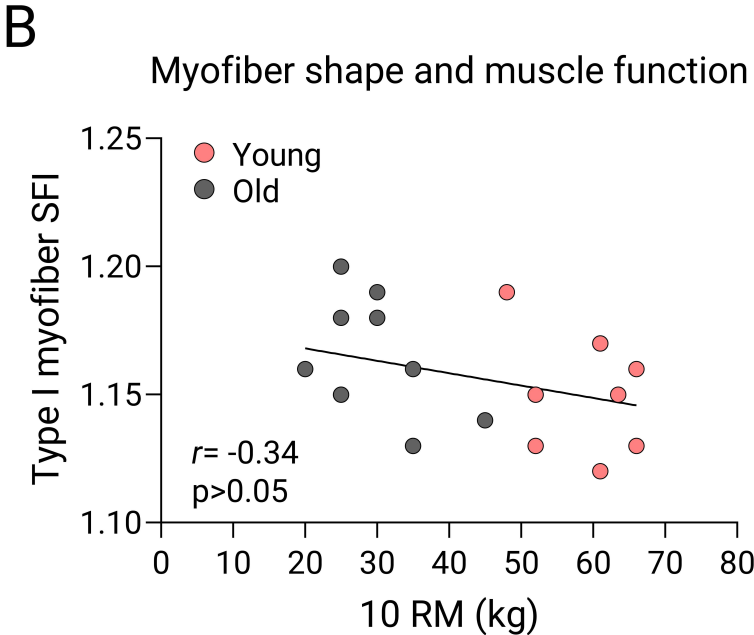

Supplement: Supplementary file 2 — Figure S2. Correlations. Correlational analyses between muscle function and type I myofibre size (a) and shape (b). Young, n = 10; and old, n = 11. Some data points (n = 4) in (b) are masked owing to overlap. Abbreviations: RM, repetition maximum; SFI, shape factor index. [file EPH-110-277-s002.pdf]
