# Supplementary material for: Ageing leads to selective type II myofibre deterioration and denervation independent of reinnervative capacity in human skeletal muscle
Source: Exp Physiol. 2024 Oct 28;110(2):277–92. doi: 10.1113/EP092222 (PMC11782179; doi:10.1113/EP092222)
Supplement: Supplementary file 3 — Table S1. Fibre size variation in muscle cross‐sections from young and older adults. [file EPH-110-277-s003.docx]

**Supplemental tables**

**Supplemental table 1**. Fibre size variation in muscle cross-sections from young and older adults

|  | **Young** | **Old** | **p value** |
| --- | --- | --- | --- |
| Fibre size variation, CV% |  |  |  |
| Mixed myofibres | 19.6 ± 4.9 | 23.6 ± 4.2 | 0.0076 |
| Type I myofibres | 21.4 ± 4.6 | 23.5 ± 4.7 | ns |
| Type II myofibres | 17.8 ± 4.6 | 23.8 ± 3.8 | 0.0053 |

CV; Coefficient of variation
